# Traditional Chinese medicine combined with conventional therapy for female kidney stone: A protocol for systematic review

**DOI:** 10.1097/MD.0000000000019611

**Published:** 2020-03-27

**Authors:** Hong Du, Yi Lei, A-Ni Gao, Xiao-Min Sun, Rui Song, Xu-Dong Yu, Sheng Deng, Hong-Mei Si, Jia Chen

**Affiliations:** a The Second Affiliated Hospital of Shaanxi University of Chinese Medicine, Xianyang, Shaanxi; b Graduate School of Beijing University of Chinese Medicine; c Department of Andrology, Dongzhimen Hospital, Beijing University of Chinese Medicine, Beijng, China.

**Keywords:** kidney stone, protocol, randomized controlled trial, traditional Chinese medicine

## Abstract

**Introduction:**

Kidney stone is caused by abnormal accumulation of crystalline substances in the kidneys. Kidney stone is one of the urinary system diseases with a high incidence. In this study, we will use the research method of randomized controlled trials to explore the effects of Traditional Chinese medicine combined with western medicine on renal function and urine metabolism in women with kidney stones. We hope that the results of this study will provide more evidence-based medical evidence for TCM to treat kidney stones, and also provide patients with more treatment options.

**Methods/design:**

This pragmatic randomized controlled trial will recruit 100 patients who are diagnosed with kidney stone. Simple randomization to conventional drug treatment with a 1:1 allocation ratio will be used. The participants will continue to receive ESWL treatment and TCM therapy. The selection of outcomes will be evaluated by the overall effectiveness of clinical efficacy.

**Discussion:**

This trial may provide evidence regarding the clinical effectiveness, safety, and cost-effectiveness of Traditional Chinese medicine for patients with Female kidney stone.

## 1 Introduction

Kidney stone is caused by abnormal accumulation of crystalline substances (such as calcium, oxalic acid, uric acid, cystine, etc.) in the kidneys.^[1]^ Kidney stone is one of the urinary system diseases with a high incidence. They are mostly caused by the long-term precipitation of water-insoluble substances in the urine and their retention in the kidneys. Forty percent to 75% of patients with kidney stone has different degrees of low back pain.^[2,3]^ The stones are large and the mobility is small, manifested as discomfort of soreness in the waist, or dull or dull pain when physical activity increases. Colic caused by small stones, sudden abdomen-like pain in the abdomen and abdomen often occurs suddenly, showing paroxysmal. Stone can occur in any part of the urinary system but often originate in the kidney.^[3]^ When kidney stone is formed, it is usually located in the renal pelvis or sacral cavities, which can be discharged into the ureter and bladder. Almost all ureteral stones come from the kidney. With the development of social economy and changes in dietary habits, the incidence of kidney stone has become higher and higher in recent years.^[4,5]^ The incidence of this disease is more men than women, but epidemiological investigations have shown that the incidence of kidney stones in women is on the rise.

For the treatment of this disease, Extracorporeal Shock-wave Lithotripsy (ESWL) is commonly used for kidney stones, and it is supplemented with symptomatic treatment such as antispasmodic, analgesic and anti-inflammatory.^[6]^ However, the effect of urinary stone excretion is not good, and serious complications such as urinary tract obstruction, hydronephrosis and abnormal urine metabolism are prone to occur after surgery. This disease belongs to the category of “*Shilin*” in Traditional Chinese medicine (TCM). TCM believes that the disease is located in the kidney and bladder, and is closely related to the liver and spleen.^[7,8]^ Most of them are bet on the bladder by moist heat, and the impurities in the urine become stones for a long time. In the long-term medical practice, the effect of TCM on kidney stones has been gradually recognized. TCM has been widely used in the treatment of kidney stones due to its safety and economic advantages.^[9]^ At present, Chinese medicine Paishi Decoction has become a classic prescription for treating kidney stones. However, there are few studies on the combination of TCM and ESWL in the treatment of kidney stones. At the same time, we found in previous literature searches that there is no research on kidney stones in women.^[10]^ Therefore, in this study, we will use the research method of randomized controlled trials to explore the effects of TCM combined with western medicine on renal function and urine metabolism in women with kidney stones. We hope that the results of this study will provide more evidence-based medical evidence for TCM to treat kidney stones, and also provide patients with more treatment options.

## 2 Methods/design

### 2.1 Study design and settings

**Figure 1:**
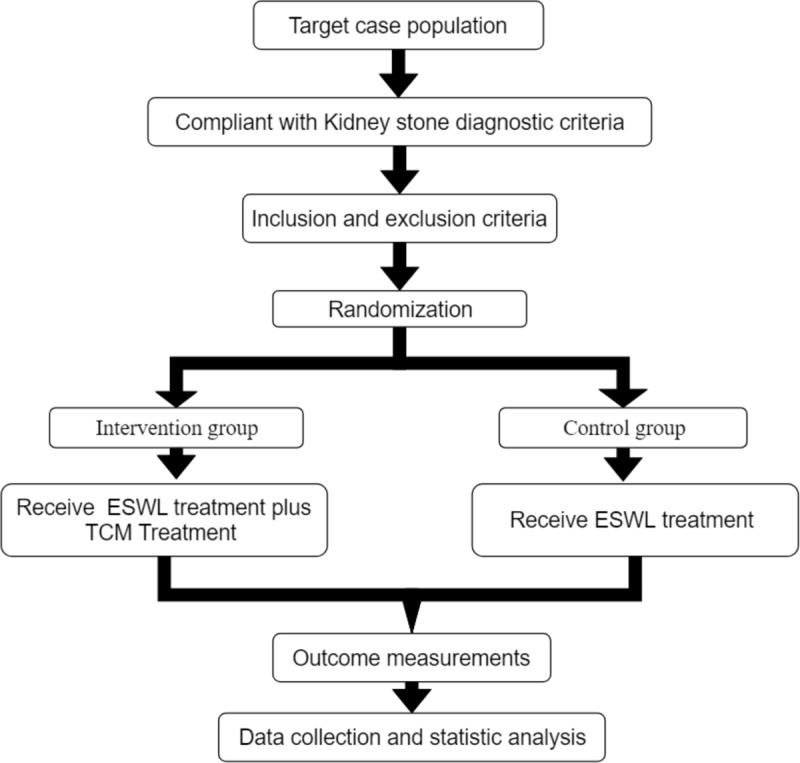
Study design flow chart.

A brief flowchart of the entire study is shown in Figure 1. We will perform a 2-group, randomized, single-blind, placebo-controlled, multi-center trial that will evaluate the efficacy and safety of acupuncture for patients with kidney stone. This study will use a completely random grouping and parallel control observation design method. We will ensure the balance of the baseline data of the 2 groups through a sufficient sample size and a completely randomized grouping method. This study will be approved by the Ethics Committee of The Second Affiliated Hospital of Shaanxi University of Chinese Medicine. We will not begin recruiting at other centers in the trial until local ethical approval has been obtained. This study is registered at http://www.chictr.org.cn/showproj.aspx?proj=49522(ChiCTR2000029860). The protocol includes elements recommended in the Standard Protocol Items: Recommendations for Interventional Trials checklist (Additional file 1).

### 2.2 Setting and participants

#### 2.2.1 Diagnostic criteria

Western medicine diagnosis refers to “*Chinese Urological Disease Diagnosis and Treatment Guide*” and “*Urinary Surgery*” in kidney stone related diagnostic standards. The diagnosis of TCM refers to the “*Guiding Principles for Clinical Research of New Chinese Medicines*”, and the syndrome is identified as damp-heat-stasis syndrome type.

#### 2.2.2 Inclusion criteria

This study will be conducted in China. Patients will be recruited from Urology/Andrology departments of The Second Affiliated Hospital of Shaanxi University of Chinese Medicine. We will enroll participants based on the following inclusion criteria:

1. Those who meet the above diagnostic criteria; those with varying degrees of hematuria, urgency, and dysuria;
2. Ultrasonography or X-ray examination support the diagnosis, visible stone parts; stone diameter ≤8 mm;
3. Female patients aged 18 to 80 years old;
4. Voluntary signing of informed consent.

### 2.3 Randomization, allocation concealment, and blinding

The web-based online randomization system to be used in this trial was provided by an independent academic data management center at The Second Affiliated Hospital of Shaanxi University of Chinese Medicine. The attending Physician will identify eligible patients according to the inclusion and exclusion criteria. Informed consent will be taken by the attending physician, and participants will be referred to a research coordinator who will randomly assign them to the intervention or control arm. The randomization list is kept by the biostatistician and research coordinator until the end of the study to ensure allocation concealment; therefore, the data analysts will be kept blinded to the allocation. The participants will be instructed not to disclose the allocation to the attending physician.

### 2.4 Interventions

Control group: We will give patients ESWL treatment. Using external shock wave lithotripsy, maintaining a voltage of 6.0 to 9. 0 kV, a frequency of 0.8 to 1.0 s/time, 1200 to 1500 times per shock, and 0.5 to 1 hour per treatment time. All patients will be treated twice, with an interval of 5 days between each treatment. Postoperative patients will receive conventional treatments such as hemostasis, anti-infection, and antispasmodic treatment. During the treatment of patients, intake of high-fat, high-calorie, high-purine, high-uric acid foods will be prohibited, and plenty of water is maintained.

Intervention group: Patients in the intervention group will be treated with Chinese herbal medicine-*Paishi* Decoction from the first day after the lithotripsy. The specific steps are as follows: take Chinese herbal medicine *Baimaogen 30 g*, *Huangqi 30 g*, *Jinqiancao* 50 g, *Haijinsha 20 g*,*Zexie, Houpo, Niuxi, and Shiwei 15 g* each, *Tusizi, Baishao, Xuduan, Cheqianzi and Yujin 10 g* each, *Gancao 6 g*. Mix the above Chinese herbs together and add 500 ml of water to cook. After filtering it, take the remaining liquid, take it orally twice a day, and take it continuously for 2 weeks. During the treatment of patients, intake of high-fat, high-calorie, high-purine, high-uric acid foods is prohibited, and plenty of water is maintained.

### 2.5 Data collection

The study data collection process is outlined in Table 1.

**Table 1:**
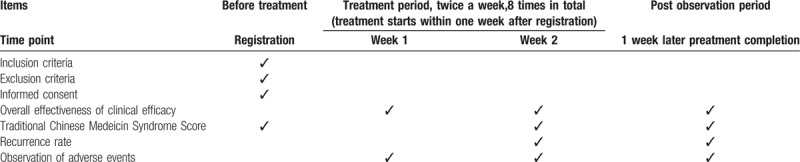
Treatment schedule and outcome measures.

### 2.6 Primary outcomes

We will compare the overall effectiveness of clinical efficacy between the 2 groups as the primary outcome measure. The clinical curative effect of the 2 groups will be evaluated with reference to the “*Guidelines for Clinical Research of New Chinese Medicines*”. The clinical symptoms and signs disappeared, and no hematuria was detected by routine urine tests, and all lithotripsy was discharged. Ultrasound showed that no residual stones were cured. Symptoms and signs were significantly improved. Hematuria was improved by routine urine tests, and ultrasound showed that some stones were effectively discharged. No change, no improvement in hematuria by routine urine test, and ultrasound showed that stones were not discharged as invalid. Total effective rate = (number of cured cases + number of effective cases) / total number of cases × 100%.

### 2.7 Secondary outcomes

The second outcome measure is based on TCM syndrome evaluation criteria.

1. Healing: The clinical symptoms and signs of TCM disappear or almost disappear, and the syndrome score is reduced by≥90%;
2. Significant effect: The clinical symptoms and signs of TCM are obviously improved, and the syndrome score is reduced by≥60%;
3. Effective: Chinese medicine Clinical symptoms and signs have improved, syndrome scores decreased by <60%, but≥30%;
4. Invalid: The clinical symptoms and signs of TCM were not improved, even worse, and the syndrome score was reduced by <30%. Integral variation formula (Nimodipine method: [(pretreatment score - post-treatment score) ÷ pretreatment score] × 100%.

### 2.8 Statistical consideration

#### 2.8.1 Sample size

The sample size is an element in choosing the test statistic. According to the sampling distribution theory, under the condition of large samples, if the population is normally distributed, the sample statistics follow the normal distribution; if the population is non-normal distribution, the sample statistics gradually follow the normal distribution. In clinical trial research, both the experimental group and the control group need to have a certain number of subjects. This is because there are variations in the experimental effects of the same experimental treatment on different subjects. The results of only one experimental observation or the experimental effects of a single subject do not explain the problem. A certain number of repeated observations are required to show the true and objective regularity of the study as a whole, and an objective estimation of the sampling error can be made. Generally speaking, the more repeated observations, the smaller the sampling error, and the higher the reliability of the observation results.

#### 2.8.2 Analysis set

The Full Analysis Set will be used for all primary analysis. Per Protocol Set analysis will be performed for evaluating the sensitivity of results.

#### 2.8.3 Statistical analysis

Data management uses EXCEL software to build a database, double entry, check for outstanding values, and lock. Statistical analysis will be performed using SPSS 25.0 software for statistical analysis. The normality of the measurement data is tested. The data obeying the normal distribution is Student's t test, which is expressed by mean ± standard deviation. The data not obeying the normal distribution is rank sum test. And marginal homogeneity test; count data are expressed by rate and composition ratio, and comparison is performed by chi-square test; repeated measurement data are expressed by mean ± standard deviation, intra-group comparison is performed by analysis of variance of repeated measurement data, and inter-group comparison is by multivariate analysis of variance (MANOVA). *P* ≤ .05 indicates that the difference is statistically significant.

### 2.9 Quality control and trial management

The management structure will comprise the principal investigator (PI), a trial management group, and a data monitoring committee. The trial management group will be responsible for conducting the trial and will meet monthly to discuss the trial progress. The PI will visit each collaborative hospital for face-to-face meetings and to share information to promote patient recruitment. The data monitoring committee will review safety and efficacy data. All data will be monitored every month through a central monitoring method. Additional monitoring may be performed at the discretion of the monitoring manager. The data monitoring committee have met once prior to the start of patient recruitment. At least twice per year, participating investigators, research assistants, and research nurses will be required to attend a training workshop on clinical research to ensure strict adherence to the study protocol and familiarity with the trial administration process. The data collected in this trial will comprise information recorded in case report forms and questionnaires. Data quality will be checked regularly by research assistants and overseen by monitors; all modifications will be marked on case report forms and data managers will recheck the data before they are officially logged. The database will be locked after all data have been cleaned. If participants withdraw from the trial during the study period, the reasons will be documented and the dropout rate will be statistically analyzed.

## 3 Discussion

At present, ESWL has been commonly used in clinical practice to crush the stones in patients with kidney stone after extracorporeal shock wave focusing, and make them excreted through the urine.^[11]^ This kind of treatment can alleviate the patient's pain to a certain extent and improve the clinical efficacy. However, ESWL is ultimately an invasive treatment. TCM classifies kidney stones as “back pain” and “stone leaching”. TCM believes that the pathogenesis is damp-heat betting and water-heat interpenetration.^[12]^ The principle of diuretic and stone-elimination should be followed during treatment. Clinical studies have shown that Chinese herbal medicine can promote the discharge of broken stones in patients. Related research results suggest that oral Chinese herbal medicine combined with ESWL in the treatment of kidney stone can effectively alleviate the clinical symptoms of patients, promote patients to row stones, and improve the treatment effect.^[13]^ Its possible mechanism is that Chinese herbal medicine can enhance ureteral peristalsis and promote the movement of stones in the ureter. On the other hand, it may be through diuretic effect to increase the internal pressure of the renal pelvis and promote the excretion of stones.^[14,15]^ In recent years, with the widespread application of ESWL, its damage to renal function and normal kidney tissue has caused widespread concern. The physical effects of shock waves and free radicals are considered to be important causes of kidney injury in patients with kidney stones.^[16]^ Therefore, exploring effective postoperative stone-assisting therapy has become a hot spot in clinical research. At the same time, we found in previous literature searches that there are no studies on kidney stones in women. Therefore, in this study, we will use the research method of randomized controlled trials to explore the effects of TCM combined with western medicine on renal function and urine metabolism in women with kidney stones. We hope that the results of this study will provide more evidence-based medical evidence for traditional Chinese medicine to treat kidney stones, and also provide patients with more treatment options.

## Acknowledgments

The authors would like to thank all the trial participants. The authors are grateful for the support for this study: trial coordinating team, surgical staff, nurses, and research departments.

## Author contributions

HD, YL, JC, SD, and AMG designed the study protocol and drafted the manuscript. XMS and XDY reviewed the study protocol and drafted the manuscript. SD and HMS is responsible for the statistical design and analysis as trial statistician. All authors carefully read and approved the final version of the manuscript.
